# Adoption of Clinical Decision Support in Multimorbidity: A Systematic Review

**DOI:** 10.2196/medinform.3503

**Published:** 2015-01-07

**Authors:** Paolo Fraccaro, Mercedes Arguello Casteleiro, John Ainsworth, Iain Buchan

**Affiliations:** ^1^NIHR Greater Manchester Primary Care Patient Safety Translational Research CentreInstitute of Population HealthThe University of ManchesterManchesterUnited Kingdom; ^2^Centre for Health InformaticsInstitute of Population HealthThe University of ManchesterManchesterUnited Kingdom; ^3^Health eResearch CentreThe University of ManchesterManchesterUnited Kingdom; ^4^Bio-Health Informatics GroupSchool of Computer ScienceThe University of ManchesterManchesterUnited Kingdom

**Keywords:** decision support systems, management, systematic review, multiple chronic diseases, multiple pathologies, multiple medications

## Abstract

**Background:**

Patients with multiple conditions have complex needs and are increasing in number as populations age. This multimorbidity is one of the greatest challenges facing health care. Having more than 1 condition generates (1) interactions between pathologies, (2) duplication of tests, (3) difficulties in adhering to often conflicting clinical practice guidelines, (4) obstacles in the continuity of care, (5) confusing self-management information, and (6) medication errors. In this context, clinical decision support (CDS) systems need to be able to handle realistic complexity and minimize iatrogenic risks.

**Objective:**

The aim of this review was to identify to what extent CDS is adopted in multimorbidity.

**Methods:**

This review followed PRISMA guidance and adopted a multidisciplinary approach. Scopus and PubMed searches were performed by combining terms from 3 different thesauri containing synonyms for (1) multimorbidity and comorbidity, (2) polypharmacy, and (3) CDS. The relevant articles were identified by examining the titles and abstracts. The full text of selected/relevant articles was analyzed in-depth. For articles appropriate for this review, data were collected on clinical tasks, diseases, decision maker, methods, data input context, user interface considerations, and evaluation of effectiveness.

**Results:**

A total of 50 articles were selected for the full in-depth analysis and 20 studies were included in the final review. Medication (n=10) and clinical guidance (n=8) were the predominant clinical tasks. Four studies focused on merging concurrent clinical practice guidelines. A total of 17 articles reported their CDS systems were knowledge-based. Most articles reviewed considered patients’ clinical records (n=19), clinical practice guidelines (n=12), and clinicians’ knowledge (n=10) as contextual input data. The most frequent diseases mentioned were cardiovascular (n=9) and diabetes mellitus (n=5). In all, 12 articles mentioned generalist doctor(s) as the decision maker(s). For articles reviewed, there were no studies referring to the active involvement of the patient in the decision-making process or to patient self-management. None of the articles reviewed adopted mobile technologies. There were no rigorous evaluations of usability or effectiveness of the CDS systems reported.

**Conclusions:**

This review shows that multimorbidity is underinvestigated in the informatics of supporting clinical decisions. CDS interventions that systematize clinical practice guidelines without considering the interactions of different conditions and care processes may lead to unhelpful or harmful clinical actions. To improve patient safety in multimorbidity, there is a need for more evidence about how both conditions and care processes interact. The data needed to build this evidence base exist in many electronic health record systems and are underused.

## Introduction

### Background

Patients affected by multiple diseases are acknowledged to be one of the greatest challenges for modern health care, especially as populations age [1]. Different terms have been used in the medical literature to refer to coexistent pathologies; the most accepted are [2] *comorbidity*, defined in 1970 as “any distinct additional clinical entity that has existed or may occur during the clinical course of a patient who has the index disease under study” [3], and *multimorbidity*, later defined as “the coexistence of 2 or more chronic conditions, where 1 is not necessarily more central than others” [4]. In this review, we look at the presence of simultaneous medical conditions as the decision-making context without emphasizing the prominence of any 1 condition, and we follow the European General Practice Research Network, which defines multimorbidity as “any combination of chronic disease with at least 1 other disease (acute or chronic) or bio-psychosocial factor (associated or not) or somatic risk factor” [5]. Here we use multimorbidity in a broad sense to infer comorbidity as well.

### Impact of Multimorbidity on Public Health

Estimates of the prevalence of multimorbidity emanate from countries with detailed primary care records. A national population study carried out in the Netherlands estimated an overall prevalence of 29.7%, ranging from 10% in those younger than 20 years to 78% in those older than 80 years [6]. Another population study in Scotland found an overall prevalence of 23.2% [7]. The prevalence of multimorbidity in a population increases with age [8]. Thus, a growing proportion of the population is affected by multimorbidity as populations age [9], particularly in countries with demographic patterns like the United Kingdom [10]. Previous studies [11-13] most commonly reported the following disease groups as likely to concur: cardiovascular diseases, diabetes mellitus, chronic kidney disease, chronic musculoskeletal disorders, chronic lung disorders, and mental ill health (particularly dementia and depression). There is also a greater burden of multimorbidity at younger ages (younger than 65 years) in deprived areas [7]. Thus, the public health and economic impact of multimorbidity is large [14]. In the United States, 84% of total health expenditure involves patients with more than 1 condition [15], whereas multimorbid patients in England account for the majority of primary care encounters [16] and this is expected to rise [15].

### Patient-Centered Care and Iatrogenic Risks

The model of care in multimorbidity is changing, from a disease- and organization-centered approach [3] to patient-centered holistic care [17]. Patient-centeredness considers psychological and physiological needs, the patient’s concerns and priorities for care, self-care, and coordination between different professions and organizations, with primary care as an integrator [17]. Although patient-centered care is ideal for managing complex, chronic conditions, it is challenging to implement [5]; therefore, at present, patients with multimorbidity are commonly underserved by poorly integrated care systems [18,19]. This fragmentation reduces the safety, effectiveness, and efficiency of care [1]. A previous study reported that 10% to 20% of unscheduled care among older multimorbid adults is iatrogenic (eg, medication-related harm) [20].

### Self-Management and Continuity of Care

The presence of simultaneous care plans for multiple conditions leads to confusion and, in turn, generates safety hazards. Clear care plans, blending clinical care with self-management, are essential in multimorbidity [21]. Such plans need to incorporate not only biomedical but also psychosocial factors, such as mood, informal care network, and patient income/finances [21]. Communication between patients/carers and health professionals over complex care plans can be challenging; therefore, self-care may be unreliable [21,22]. For example, it was estimated an average Medicare patient in the United States with 1 chronic condition sees 4 different health care professionals in 1 year and this number increases to 14 in the presence of 5 different chronic conditions [22]. Increasing the number of health professionals involved creates a combinatorial explosion of communication interfaces and, for the patient, greater difficulty in understanding, remembering, and recalling guidance [22]. The most common problems arising from this miscommunication are duplication of tests and harmful decisions made on the basis of incomplete or incorrect information [23,24]. Primary care and general practitioners, in particular, are seen as a nexus of coordination for complex care such as this [24]. However, general practitioner workload is increasing beyond its capacity with the rising prevalence of chronic diseases and multimorbidity [25].

### Clinical Practice Guidelines and Polypharmacy

Clinical research processes tend to focus narrowly on a single disease, mechanism, or treatment. This parsimony is reflected in the production of clinical practice guidelines; therefore, interactions between diseases are barely touched upon in care pathways (even if they are referred to as “integrated”) [26]. More recently, organizations such as the National Institute for Health and Care Excellence (NICE) have started to address multimorbidity explicitly [27] and a framework of principles for system-wide action to deal with comorbidities has been developed in England by the Department of Health and the National Health Service (NHS) [28]. Most current guidelines, however, do not consider interactions between diseases or between treatments [29]. Therefore, potential synergies or conflicts between different care pathways operating for the same patient may be missed [30]. For example, Boyd et al [29] applied clinical practice guidelines to a hypothetical case of a 79-year-old woman with multiple moderately severe chronic conditions (osteoporosis, osteoarthritis, diabetes mellitus, hypertension, and chronic obstructive pulmonary diseases). The guideline-derived treatment regimen was extremely complex and potentially harmful—comprising 14 nonpharmacologic treatments (ie, self-monitoring, diet, exercise, health care visits, and laboratory testing) and 12 unique medications with 19 doses of medication per day [29]. Even in simpler cases, such as the presence of 2 diseases and 2 related treatments, researchers report 16 possible exposure patterns (half relevant for clinical practice guidelines) and 4 possible interaction combinations [26]. The 2 previous examples precipitate a “prescribing cascade” whereby drugs are prescribed to treat the adverse effects of other drugs, which is common in polypharmacy (the use of multiple medications) [31].

Even the most primary care-focused of health care systems, such as the NHS [27], do not deal safely, effectively, or efficiently with multimorbidity and polypharmacy [32]. In the future, with an aging population, most health care system resources will be stretched by the care needs of multimorbid patients [33].

### Informatics Implications

Multimorbid health care requires complex communication, analysis, summarization, and presentation of heterogeneous clinical information from multiple sources. It is acknowledged that electronic health records (EHRs), especially in primary care, require enhanced functionality to support decisions in these complex care processes [34]. A clinical decision support (CDS) system provides “clinicians, patients, or individuals with knowledge and person-specific or population information, intelligently filtered or presented at appropriate times, to foster better health processes, better individual patient care, and better population health.” [35]. Despite notable failures [36], CDS systems have the potential to improve clinical outcomes [37,38]. Indeed, multimorbidity was defined as one of the “grand challenges in clinical decision support” by Sittig et al [39]; however, this area remains underinvestigated [40,41], with concerns raised over the unmet needs in primary care [40]. Some of the current challenges are lack of provision of integrated clinical practice guidelines, disease-centered rather than patient-centered approaches, difficulties in embedding CDS into clinical systems, and lack of training to make best use of CDS [40]. EHRs and computerized physician order entry systems include rules that deal with drug-drug interactions; however, the whole patient context is not considered and the system may “overalert” physicians [42]. The overalert is another main risk in multimorbidity, which is known as *alert fatigue*: “the mental state provoked by managing too many irrelevant alerts from the system, which consume physical and psychological energies and lead the user to ignore also the relevant alerts resulting in potential harm for the patient” [43]. Prescribing alerts are especially important in polypharmacy, which has well-established risks of harm [44]. However, in some situations, multiple prescriptions are valid [30] and should not be dissuaded by inappropriate alerts. Context awareness, such as an “application’s ability to adapt to changing circumstances and respond according to the context of use” [45], is crucial in decision support interventions [46], especially for multimorbidity where many variables are in place. However, a greater understanding of which information and sociotechnical factors of the context have to be taken into account in health care has still to be established [47].

Previous reviews have investigated specific aspects of CDS in multimorbidity; for example, prescribing in the elderly [48] and chronic disease management [49]. We could find no satisfactory review of CDS in multimorbidity from a technical/methodological perspective to guide the engineering of future systems. This interdisciplinary review plugs that gap.

### Aim and Objectives

The aim was to review the current state of the art of CDS in multimorbidity. The objectives were to review the aspects of decision support target, contextual information about patients/practitioners/services, decision support technology, user interface considerations, decision maker(s), diseases, and evaluation. These aspects were analyzed to identify what works and what does not in CDS for multimorbidity, why systems failed to produce the expected outcomes, and what solutions might be adopted to address the problems.

## Methods

### Overview

This review follows the guidelines from Preferred Reporting Items for Systematic reviews and Meta-Analyses (PRISMA) framework [50]. PRISMA consists of a list of 27 items and a 4-phase flow diagram to complete that was identified as the optimal way to perform and report systematic reviews and meta-analyses about health care interventions by an experienced group of researchers and methodologists [50].

### Eligibility

#### Inclusion Criteria

Studies that linked the concepts of multimorbidity, comorbidity, or polypharmacy to the concept of CDS, referring to the definitions provided previously, were selected from the literature.

The studies included in this literature review are articles about CDS systems that (1) address general issues about the multimorbid population, (2) support care for a particular subpopulation of multimorbid patients, (3) manage comorbidities related to a main disease, (4) deal with multiple concurrent medications in multimorbid population, and (5) describe statistical or machine-learning methods for clinical prediction in which the multimorbid patients’ data feed the modeling/learning and an holistic approach is adopted.

#### Exclusion Criteria

Studies excluded from this literature review were about (1) CDS characteristics in general, without describing a CDS system in detail; (2) economic evaluations of CDS; (3) CDS systems in which multimorbidity was not a key feature; (4) social and operational research into CDS with no reference to clinical outcomes; (5) statistical or machine-learning approaches in which comorbidities were part of the model, but the patient-centered approach was not considered; and (6) systems that checked drug-drug interactions by means of simple rules, without taking into account multimorbidity or comorbidities.

### Information Sources

MEDLINE and Scopus [51] were selected as the source indexes because they conform to the Cochrane requirement [52] of being “searched electronically both for words in the title or abstract and by using the standardized indexing terms, or controlled vocabulary, assigned to each record.” We used the PubMed [53] interface to MEDLINE, which also includes up-to-date citations not yet indexed in MEDLINE [52]. In addition, Scopus can use Medical Subject Headings (MeSH) terms for structured queries [54].

Some target studies could only be found in the grey literature, such as theses and conference proceedings. Scopus allows search restrictions to some categories of grey literature, such as conference proceedings. This wider searching aimed to reduce publication bias.

The searches were performed in December 2013 and January 2014 without any restriction in the publication date.

### Search

For the search, we followed 3 key points from the Cochrane Handbook [52]:

1. Searches should seek high sensitivity—this may result in poor precision.
2. Too many different search concepts should be avoided, but a wide variety of search terms should be combined with “or” within each concept.
3. Both free-text and subject headings should be used (eg, MeSH) [55].

The focused clinical question that drove this systematic review was: What is the current level of adoption of CDS in multimorbidity? To answer this question, 3 different search concepts were selected:

1. Decision support: it has many related MeSH descriptors, such as “decision support systems, management” or “decision support techniques.” Examples of individual hyponyms manually selected are “clinical decision support system,” “decision support software,” and “decision support tool.”
2. Multimorbidity: it has zero related MeSH descriptors. Semantically, the closed concept comorbidity has 1 MeSH descriptor. Examples of synonyms manually selected are “concurrent conditions,” “multiple chronic diseases,” and “multiple pathologies.”
3. Polypharmacy: it has just 1 MeSH descriptor and it should not be confused with the concept polypharmacology. Examples of synonyms manually selected are “several prescriptions,” “poly-prescriptions,” and “multiple medications.”

In essence, the search created for the focused clinical question that drove this systematic review was based on 3 different search concepts and the hyponyms and synonym terms combined with “or.” Conceptually, our clinical query was the following: *<decision support> AND (<multimorbidity> OR <polypharmacy>).*

**Figure 1:**
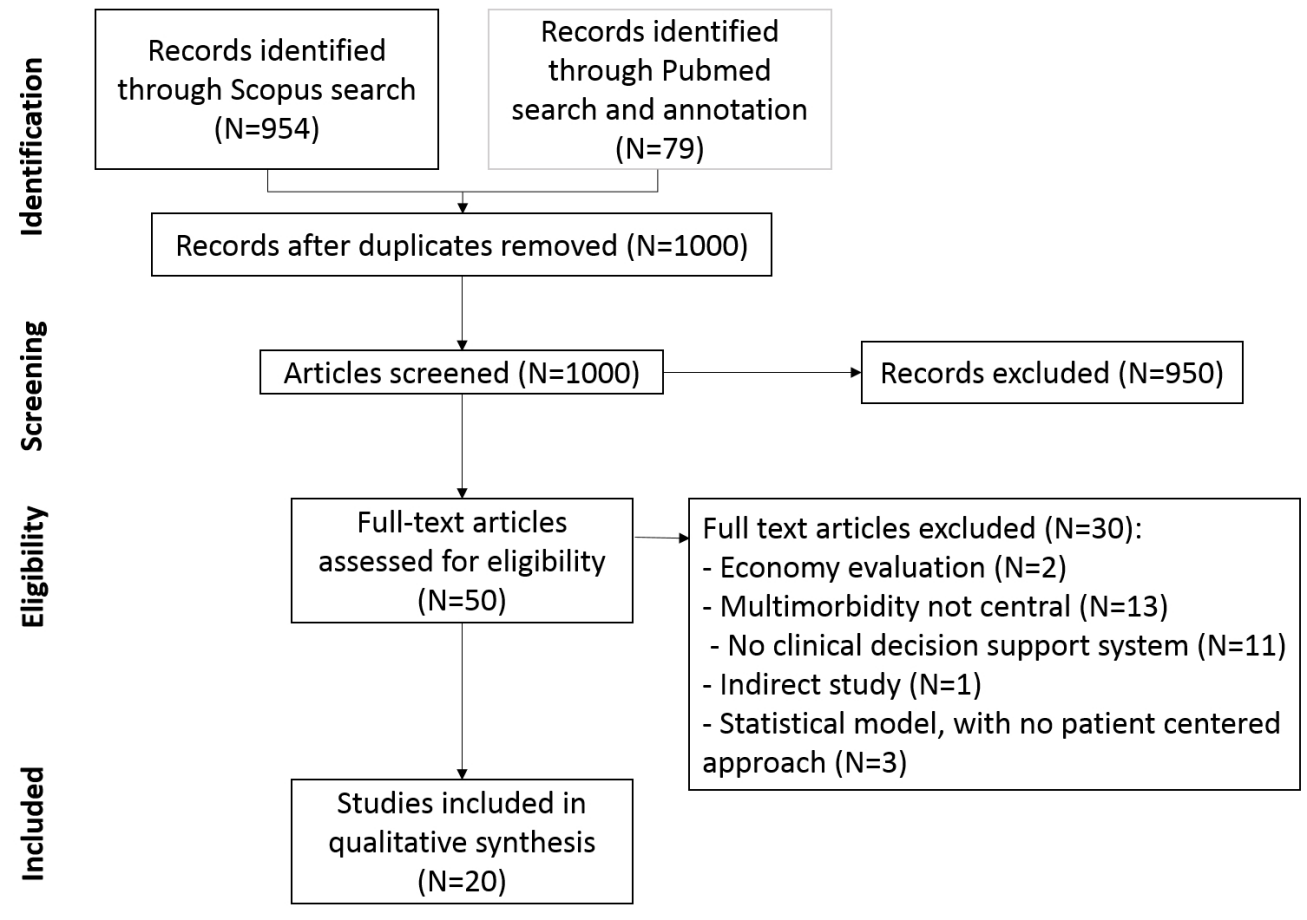
Review flow diagram.

In Scopus, the query created imposed that the relevant terms selected appear in the title, abstract, or keywords. The search yielded 954 articles (see Figure 1). Only literature from the social sciences, arts, and humanities was excluded from the search, and no restriction on the type of publication was imposed. Therefore, a wider selection of articles beyond the grey literature was retrieved.

Because multimorbidity is underrepresented in MeSH (ie, no MeSH descriptor), we created a PubMed query that looked for the relevant terms selected in the title/abstract. The search created yielded 10,223 articles (ie, 10 times more document results than in Scopus). We investigated the origin of this high number by looking at the query as it appeared under search details when using the PubMed search engine. Some of the synonyms manually selected for multimorbidity were not recognized; thus, they were split up automatically by PubMed [56]. The query as executed in PubMed contained overly general terms, such as “conditions,” “diseases,” and “pathologies.” This severely affected the performance of the query. To further illustrate this, a subquery automatically generated by PubMed as part of the original query “decision support[Title/Abstract] AND conditions[Title/Abstract]” yielded 420 results. However, this subquery did not reflect our focused clinical question and it was very unlikely that it retrieve the articles that we were interested in. Because the quality of any search depends on all constituents, we recognized that our original query was unsuitable for the PubMed search engine. More importantly, we became aware of the difficulties of constructing a PubMed query tailored to the medical question being investigated. Next, we tried to create more focused queries for the PubMed search engine, such as “multimorbidity[Title/Abstract] AND decision support[Title/Abstract],” which yielded only 6 articles. The low number of articles retrieved made us suspect that a substantial amount of articles were missing.

Knowing other researchers who were also conducting systematic reviews in the area of CDS, we thought of a search intended for a global evidence map [57] (ie, a search that sought to address broader questions about a particular area rather than focused clinical questions). Global evidence maps are similar to systematic reviews because they are both conducted in a formal process; however, the time taken for a global evidence map is longer (in excess of 2 years [58]). We were interested in decision support related to electronic clinical documentation systems and safety surveillance, so we created a new PubMed query to provide a better context of the area under study where our clinical query should focus on. The new query as it appeared under search details when using the PubMed search engine was “decision support[Title/Abstract] OR (safety[Title/Abstract] AND surveillance[Title/Abstract]) OR electronic health record[Title/Abstract] OR electronic medical record[Title/Abstract] OR electronic patient record[Title/Abstract].” This approach was adopted to guarantee the inclusion of all relevant articles even when CDS functionalities were described in studies about EHRs or safety surveillance systems without using CDS-related terms. To identify articles relevant to our focused clinical question, we used automatic annotation of all articles’ excerpts retrieved by the broader query using the hyponyms and synonym terms that appeared in the original clinical query for the 3 different search concepts originally selected. For details, see the next subsection.

### Study Selection

For the PubMed article excerpts retrieved out of the broad query, we modified the manual approach to screening citations for systematic reviews and adopted some automation. In the area of automated document classification, there is an emerging body of research that uses machine-learning methods to help with the process of citation screening (eg, [59]). We adopted a simpler, but well-founded, type of automation for prescreening PubMed article excerpts, which did not classify article excerpts as “relevant” or “irrelevant.” We used automatic annotation of text (title and abstracts) based on a controlled vocabulary known beforehand and tailored to our study. This method is analogous to the bioinformatics practice of relating genes that have been annotated using a common schema, such as an ontology [60,61], which is directly relevant to systematic reviews [62,63]. We note that the Cochrane Collaboration is considering ontologies to support evidence synthesis [62].

The annotation was performed using a controlled vocabulary (ie, the list of the hyponyms and synonym terms manually created for our clinical query). This annotation can coexist with native annotations from PubMed article excerpts based on MeSH and/or authors’ keywords. The concrete details of the annotation process are out of the scope of this paper. Once the annotation was performed, a selection of articles were selected based on our clinical query “<decision support> AND (<multimorbidity> OR <polypharmacy>).” Thus, only article excerpts with at least 1 term in title/abstract related to decision support and at least 1 term in title/abstract related to multimorbidity or polypharmacy were identified as related to our clinical question.

Articles obtained by the preceding procedure were combined with the ones from the Scopus search and, after removing duplicates, screened based on title and abstract. Relevant articles were assessed through full-text analysis to select the articles to be included in the systematic review.

### Data Collection Process and Data Items

#### Overview

A careful selection of relevant features was agreed on by the authors (PF, JA, and IB) and data on the following aspects were collected. A summary was generated for each data item and study.

#### Decision Support Target

This included clinical tasks supported by the CDS system: prevention, diagnosis, care pathway guidance (ie, management of patients according to clinical practice guidelines), medication (eg, prescription, medication review), patient education, patient self-management, and care continuity (supporting communication between health care professionals involved in multimorbid patients).

#### Contextual Information

Information was collected regarding the context processed or taken into account by the system to provide support: patient clinical notes (ie, demographics or family history), laboratory results, comorbidities, medications, clinical practice guidelines, and clinicians’ knowledge.

#### Decision Support Technology

These data included:

1. Mode of delivery: type of technical solution used to deliver the system: desktop application, Web application, and mobile application.
2. Methodology: methods used to perform the CDS intervention: data visualization techniques [64] (ie, providing intuitive interfaces to minimize errors); social network techniques; international communication and coding standard, such as Health Level Seven International (HL7) [65] to communicate information and Systematized Nomenclature of Medicine Clinical Terms (SNOMED CT) [66] or International Classification of Diseases (ICD9-10) [67], to store data; machine-learning techniques [68]; natural language processing [69]; knowledge-based systems [70] (ie, using rules or based on ontologies [71]); and mobile technologies.
3. User interface considerations: reported considerations about techniques to enhance and make easier user utilization of the system: interactivity, user-centered design, summarization, and workflow graphs.
4. Decision maker(s): user(s) of the CDS system: nurse, specialist doctor, pharmacist, generalist doctor (ie, general practitioner or family doctor), and patient.
5. Diseases/conditions: CDS target conditions: obesity, diabetes mellitus, cardiovascular diseases, chronic respiratory diseases, chronic kidney disease, neurological conditions, mental health disorders, chronic musculoskeletal diseases, etc.
6. Evaluation: type of evaluation of system’s effectiveness: uncontrolled impact studies (eg, surveys or health services measurements before/after CDS), controlled comparisons (eg, comparing new vs old/no CDS), and no evaluation.

### Synthesis of Results

The results of the review were summarized in a table. The table was organized by the aspects of CDS defined previously and provides a qualitative summary for each included study. An additional quantitative summary to highlight general trends over time and patterns of evidence is also provided.

## Results

### Study Selection

The search via Scopus retrieved 954 articles. We retrieved 17,145 articles via PubMed by using the broad search and annotation introduced previously; 79 results were recalled. After removing duplicates and screening the title and abstract, 50 articles were selected for in-depth analysis of the full text. A total of 20 studies were included in the review. The PRISMA process was followed and is reported in Figure 1.

### Study Characteristics

Table 1 contains the summary of all data items collected for each study included in the review along with its reference.

**Table 1:** Summary of collected items for included studies.

Authors	Decision support target	Contextual information	Decision support methods/delivery	User interface considerations	Decision maker/diseases	Evaluation
Abidi [72]	Pathways (merging clinical practice guidelines for different diseases into 1 personalized guideline)	Patient clinical notes & clinical practice guidelines	Knowledge-based system (ontology) & international standards/—	—	—/—	—
Abidi et al [73]	Diagnosis & pathways (alignment of care pathways in a patient-specific comorbid combination) & patient education	Patient clinical notes & clinical practice guidelines	Knowledge-based system (ontology based)/desktop application	Interactivity & summarization	Generalist doctor/chronic cardiovascular diseases	Controlled comparison-expert panel (revision by 2 generalist doctors and 1 specialist doctor)
Bindoff et al [74]	Medication (review)	Patient clinical notes & medications & laboratory results	Knowledge-based system (rule based)/ —	—	Pharmacists/—	Controlled comparison-human vs system comparison (system identified more problems)
Dassen et al [75]	Medication (prescription)	Patient clinical notes & medications & clinical practice guidelines & comorbidities & laboratory results	Knowledge-based system (ontology based) & international standards/desktop application	Interactivity & workflow graphs	Specialist doctor/cardiovascular diseases	—
de Wit et al [76]	Medication (review)	Patient clinical notes & clinical practice guidelines & clinician knowledge & laboratory results	Knowledge-based system (rule based)/—	—	Nurses/other (home care for the elderly)	No evaluation
Duke et al [77]	Medication (review)	Medications & clinician knowledge	Knowledge-based system & data visualization techniques & natural language processing/Web platform	Interactivity & summarization	Specialist doctor & generalist doctor/—	Controlled comparison-new vs old system (same accuracy but decreasing in time of 60%)
Farkas et al [78]	Diagnosis (comorbidities)	Patient clinical notes	Natural language processing/—	—	—/obesity	Controlled comparison-simulations (Fβ=1 score of 97% for classification based on textual evidence and 96% for intuitive judgments; Fβ=1 score of 76% for classification based on textual evidence and 67% for intuitive judgments)
Georg et al [79]	Medication (prescription)	Patient clinical notes & clinical practice guidelines	Knowledge-based system (rule based)/—	—	Generalist doctor/cardiovascular diseases	—
Grando et al [80]	Medication (prescription)	Patient clinical notes & clinical practice guidelines	Knowledge-based system (ontology based)/—	—	Generalist doctor/chronic respiratory diseases & diabetes & cardiovascular diseases & chronic musculoskeletal diseases & others	—
Jafarpour et al [81]	Pathways (merging clinical practice guidelines for different diseases into 1 personalized guideline)	Patient clinical notes & clinical practice guidelines & clinician knowledge	Knowledge-based system (ontology based)/—	—	Generalist doctor/cardiovascular diseases	No evaluation
Martínez-García et al [82]	Care continuity & pathways	Patient clinical notes & clinical practice guidelines & clinician knowledge	International standards & social network techniques/Web application (linked to electronic health record)	—	Nurse, generalist doctor, specialist doctor/—	Controlled comparison-survey (positively judged)
Michel et al [83]	Medication (prescription)	Patient clinical notes & clinical practice guidelines & clinician knowledge & medications & laboratory results & comorbidities	Knowledge-based system & data visualization techniques & international standards/desktop application (linked to electronic health record)	Summarization	Generalist doctor/chronic pain (opioid treated)	—
Naureckas et al [84]	Diagnosis & pathways	Patient clinical notes & clinical practice guidelines	Knowledge-based system & data visualization techniques/desktop application (linked to electronic health record)	User-centered design	Generalist doctor/child obesity and related diseases (eg, diabetes, cardiovascular diseases, chronic kidney disease)	Impact evaluation-service performance metrics & survey
Riaño et al [85]	Diagnosis & medication (prescription) & pathways (developing a personalized treatment) & prevention	Patient clinical notes & clinical practice guidelines & clinician knowledge	Knowledge-based system (ontology based) & international standards/desktop application (linked to electronic health record)	—	Generalist doctor/home care in long-term conditions (eg, obesity, diabetes, cardiovascular diseases, chronic respiratory diseases, chronic kidney disease, neurological conditions, mental health disorders, chronic musculoskeletal diseases)	Controlled comparison-survey (positively judged)
Riaño et al [86]	Medication (prescription)	Patient clinical notes & clinician knowledge	Knowledge-based system (rule based)/—	—	Generalist doctor/cardiovascular diseases & diabetes	Controlled comparison-expert panel (results validated by a generalist doctor)
Suojanen et al [87]	Diagnosis	Patient clinical notes & clinician knowledge	Machine learning/—	—	Specialist doctor/chronic neurological diseases	Controlled comparison-simulation (out of 30 cases: false positive rate=19%; false negative rate=23%)
Vallverdú et al [88]	Medication (prescription)	Patient clinical notes & clinician knowledge	Knowledge-based system (rule based)/desktop application	—	Generalist doctor/cardiovascular diseases & diabetes	Controlled comparison-expert panel (agreement with output from the system 100%-20/20)
Wicht et al [89]	Diagnosis (comorbidities)	Patient clinical notes & clinician knowledge	Knowledge-based system + data visualization techniques/Web platform	Interactivity	Specialist doctor/other (cancer)	Controlled comparison-expert panel (agreement with output from the system 84%-26/31)
Wilk et al [90]	Pathways (merging clinical practice guidelines for different diseases into 1 personalized guideline)	Patient clinical notes & clinical practice guidelines & clinician knowledge	Knowledge-based system (rule-based constraint logic programming)/—	Workflow graphs	Generalist doctor/other (duodenal ulcer, transient ischemic attack)	—
Wilk et al [91]	Pathways (alerting physicians about possible adverse interactions between 2 concurrent clinical practice guidelines)	Patient clinical notes & clinical practice guidelines	Knowledge-based system (rule-based constraint logic programming [92])/—	—	Specialist doctor & generalist doctor/chronic neurological & gastrointestinal diseases	—

### Results of Individual Studies

Table 2 shows the frequency distribution of the categories of aspects of CDS reported.

Most articles reviewed focused on 1 of 3 clinical tasks: medication (n=10), clinical guidance (n=8), and diagnosis (n=6). From a methodological point of view, knowledge-based systems were the most frequently used (n=17). To further illustrate this, Riaño et al [85] described a CDS system that targets 3 decisions and uses knowledge-based systems. The authors developed a system that (1) provided patient-centered recommendations to better manage chronic diseases in the home setting and (2) used EHRs to refine an ontology, which described relevant concepts from clinical practice guidelines and the literature for 19 chronic diseases. The goal of this study was a patient-tailored ontology that contained patient-specific concepts that could be used to verify the diagnosis entered into the system. Starting from the personalized ontology, general treatment plans and patient management instructions could be combined into an individual plan. For multimorbid patients, a semiautomatic procedure was applied that involved the system’s end-user. The system was able also to identify preventive opportunities by looking for anomalous circumstances, such as diagnosis inconsistent with other information or information missing which should always be presented alongside other information.

Abidi et al [73] presented a system that helped doctors in diagnosis and management of patients (2 decision support targets) and used an ontology (knowledge centric) that was able to align clinical pathways for the multimorbid patient.

In the articles reviewed, medication was the main theme by far. This clinical task had the most contextualized input data and appeared as prescription (n=7) and medication review (n=3). Michel et al [83] developed a system that aimed to guide the generalist doctor through a summary of comprehensive relevant information (patient information, patient medication, patient laboratory results, and patient comorbidities) and suggested the optimal opioid treat for chronic pain. Dassen et al [75] developed a system, along the lines of Michel at al [68], considering comprehensive relevant information (patient information, patient medication, patient laboratory results, and patient comorbidities) and used an ontology to support cardiologists’ prescriptions according to clinical practice guidelines. The articles by de Wit et al [76] focused on medication review and their system was intended to support safer care for the elderly. The system was capable of processing extracts of clinical data from electronic prescribing systems and EHRs (containing patient medication, patient conditions, and patient laboratory results) and alerted nurses about potentially harmful situations.

Another prevalent theme was the possible interaction between concurrent clinical practice guidelines for multimorbid patients. For example, Abidi et al [73] and Jafarpour et al [81] used ontologies to develop systems to merge 2 concurrent clinical practice guidelines into a comorbid personalized guideline. Jafarpour et al [81] carried out this task by creating an ontology that collected merging criteria obtained from clinical experts. Wilk et al [90,91] used constraint logic programming to identify and mitigate possible adverse interactions between clinical practice guidelines. One system alerted doctors about possible hazards and suggested how to mitigate them [91]. Martinez-Garcia et al [82] developed a system that improved clinical guidance by providing health care professionals with relevant information from clinical practice guidelines and also supported communication between health care professionals. Their system (1) was directly linked to the EHR through HL7, an international standard for interoperability in health care and (2) adopted social networking techniques to enhance the continuity of care through a Web platform—it provided relevant patient information and performed safety checks according to clinical practice guidelines.

Some studies addressed the diagnosis of comorbidities for patients affected by an index condition/disease. For example, Farkas et al [78] used natural language processing applied to clinical notes to diagnose comorbidities in obese patients. Suojanen et al [87] used machine learning (causal Bayesian networks) for diagnosis of multiple concurrent neuropathies.

**Table 2:** Synthesis of occurrences’ numbers and references for collected data items.

Theme and category		Frequency	References
**Decision support task**			
	Prevention	1	[85]
	Diagnosis	6	[73,78,84,85,87,89]
	Pathway	8	[72,73,81,82,84,85,90,91]
	Medication	10	[74-77,79,80,83,85,86,88]
	Patient education	1	[73]
	Continuity of care	1	[82]
	Self-management	0	—
**Decision support technology**			
	Data visualization techniques	4	[77,83,84,89]
	Social network techniques	1	[82]
	International standards	5	[73,75,82,83,85]
	Machine learning	1	[87]
	Natural language processing	2	[77,78]
	Knowledge-based system	17	[72-77,79-81,83-86,88-91]
	Mobile technologies	0	—
**Contextual information**			
	Patient clinical notes	19	[72-76,78-91]
	Laboratory results	4	[74-76,83]
	Comorbidities	2	[75,83]
	Medications	4	[74,75,77,83]
	Clinician knowledge	11	[76,77,81-83,85-90]
	Clinical practice guidelines	13	[72,73,75,76,79-85,90,91]
**Decision maker(s)**			
	Nurse	2	[76,82]
	Specialist doctor	6	[75,77,82,87,89,91]
	Generalist doctor	13	[73,77,79-86,88,90,91]
	Pharmacist	1	[74]
	Patient	0	—
	Not specified	2	[72,78]
**Diseases**			
	Obesity	3	[78,84,85]
	Diabetes	5	[80,84-86,88]
	Cardiovascular diseases	9	[73,75,79-81,84-86,88]
	Chronic respiratory diseases	2	[80,85]
	Chronic kidney diseases	2	[84,85]
	Chronic neurological conditions	3	[85,87,91]
	Mental health disorders	1	[85]
	Chronic musculoskeletal diseases	2	[80,85]
	Other	8	[76,80,83-85,89,90]
	Not specified	4	[72,74,77,82]

**User interface considerations**			
	Interactivity	4	[73,75,77,89]
	User-centered design	1	[84]
	Summarization	3	[73,77,83]
	Workflow graphs	2	[75,90]
	Not specified	13	[73,74,76,78-82,85-88,91]
**Evaluation**			
	Impact evaluation (service performance metrics)	1	[84]
	Impact evaluation (survey)	1	[84]
	Controlled comparison (expert panel)	4	[72,86,88,89]
	Controlled comparison (survey)	2	[82,85]
	Controlled comparison (simulation)	2	[78,87]
	Controlled comparison (human vs system)	1	[74]
	Controlled comparison (new vs old system)	1	[77]
	No evaluation	2	[76,81]
	Not specified	7	[72,75,79,80,83,90,91]

For the decision makers, generalist doctors were the most cited users of the CDS systems (n=13) followed by specialist doctors (n=6). No articles reported the patient as the decision maker. The system that appeared to involve the largest number of decision makers was described by Martinez-Garcia et al [82], in which nurses, specialist doctors, and generalist doctors were end users.

For disease, many articles considered multiple diseases (eg, [80,84-86,91]), with Riano et al [85] reporting 19 chronic conditions.

For user interface considerations, most articles (n=13) did not provide details about the user interface. Where this information was provided, interactivity (n=4) [73,75,77,89] and summarization (n=3) [73,77,83] were the most cited features, whereas workflow graphs [75,90] were seldom mentioned. Only Naureckas et al [84] presented a CDS system that adopted a user-centered design with prompts and forms that helped generalist doctors to develop more effective behaviors for supporting diagnosis, management, and screening of comorbidities for children with obesity.

Regarding type of evaluation, some articles reported effectiveness objectively, including controlled comparisons (n=9) or impact evaluations (n=1). The articles that conducted surveys about their systems achieved positive judgments about the outcome provided [82,85]. In terms of accuracy, many studies reported good performance [87-89]. Duke et al [77] compared UpToDate [93] with a new system that had the same accuracy, but improved (by 60%) timeliness of decision. Bindoff et al [74] compared a CDS system with expert pharmacists when performing a medication review; overall, the system identified more potential problems than the human experts did.

## Discussion

### Summary of Evidence

#### Overview

This literature review found a modest number of articles addressing CDS and multimorbidity—an evidence base disproportionately small in comparison to the need for decision support.

#### The Lack of Patient-Centered Approaches

Most articles dealt with CDS targets that (1) were narrowly defined in terms of comorbidities around an index condition or (2) considered patient comorbidities only during prescription for a specific condition. Thereby, only a few of the studies reviewed referred to multimorbidity using a patient-centered approach, which is the ideal [5]. Riano et al [85] adopted a comprehensive approach to integrated care; however, user intervention is necessary to personalize treatments when multimorbidity is present.

#### Combination of Clinical Practice Guidelines

An important challenge of multimorbidity in CDS is the combination of clinical practice guidelines in a nonharmful way [39]. We found some studies that addressed this explicitly. An interesting solution was introduced by Jafarpour et al [81], which created an ontology with merging criteria provided by experts. Although rigorous evidence is lacking, to exploit physicians’ “clinical mind-lines,” such as “tacit guidelines that are internalized and collectively reinforced from the experience and discussion with colleagues and patients to embody the complex and flexible knowledge needed in practice” [94], seems the only solution. However, all systems described in the articles reviewed tended to simplify the analysis by referring to only 2 concurrent clinical practice guidelines. This scenario is too simplistic for the current reality because multimorbid patients often face more than 2 simultaneous pathologies [29].

#### Continuity of Care

Discontinuity of care between different health professionals is an important source of safety problems, which is highly relevant to multimorbidity considering the large numbers of professionals involved. Yet only 1 article [82] considered this aspect. Prevalent technologies such as social media may foster communication across different clinical settings. There is a notable gap in the evidence base here.

#### No Self-Management Interventions

Self-management is key in multimorbidity [21]. In the articles reviewed, no CDS interventions for multimorbid patient self-management were found. Similarly, we noticed the absence of mobile technologies for CDS in multimorbidity.

#### Methodological Considerations

From a methodological point of view, knowledge-based systems were most commonly reported. Data-driven methods, such as machine-learning techniques, were barely used in the reviewed studies, with just 1 study adopting them [87].

#### The Technological Interoperability Shortfalls

Multimorbidity is composed of interacting variables; therefore, systems need to be aware of as many contextual factors as possible to deliver relevant support and information [95]. Emerging international standards, such as HL7, are supposed to enable interoperability in health care; however, only 1 article reviewed used HL7, the system developed by Martinez-Garcia et al [82].

#### The Need for More Rigorous Evaluations

Evaluations of usability and effectiveness of systems are key to avoiding patient harm and waste in health care systems [96]. The so called “e-iatrogenesis” [97] arising from information systems has more potential pitfalls when there are multiple conditions. Rigorous evaluations are needed to test systems before and after their deployment to guarantee patient safety [98,99]. We found a lack of rigorous evaluations of effectiveness and usability here, which is consistent with the overall state of CDS [36] research. Patient safety needs to be assured by rigorous evaluation, not only of the underlying software/technologies but also of their real-world interaction with users [100]. The expected approaches to evaluating human-computer interaction [101,102] were not found in the articles we examined.

### Limitations

This review has several limitations. First, only Scopus and PubMed sources were searched—other relevant material may exist in the grey literature. Second, the titles and abstracts of the articles selected are anchored to the terms included in the 3 thesauri—some articles may have been missed if other synonyms were used. Third, it was not possible to find studies covering all aspects of CDS we considered—some aspects, such as the evaluation of the effectiveness and usability, were quite sparsely covered, but this is a general weakness of the CDS literature [36]. Finally, we did not follow the traditional systematic review process for all searches. However, we are confident that our strategy guaranteed the inclusion of all relevant articles about the topic. There is an ongoing discussion of what should and should not be automated in systematic reviews, particularly to strike the right balance between depth and timeliness [103]. Here we took the middle ground, using computational methods to make a more “concept-complete” search tractable. Therefore, this review may contribute to the ongoing discussion about semiautomated prescreening of medical literature while preserving rigorous methods of evidence synthesis.

### Implications for Future Research and Conclusions

This review shows how multimorbidity is understudied in CDS, yet this is an area of public health and clinical importance that should be a prime target for CDS research.

There are already many technologies in health care and industry relevant to dealing with the complexity of multimorbid decision support. Kawamoto et al [104] argue that wider adoption of international terminologies (eg, SNOMED CT) and electronic health record standards can lead to better CDS, tapping into the vast amount of data produced in routine clinical practice for multimorbid patients. Moreover, technical frameworks [105] were already proposed for a “shared and informed decision making” in industry that with appropriate adjustments could be used to enhance continuity of care in multimorbidity. In addition, the absence of any substantial articles dealing with self-care for people affected by multiple conditions was remarkable given the rapid growth in connected/consumer health and its inevitable influence on CDS in the future.

Multimorbidity is a relatively new field of clinical research and more evidence is needed to support CDS in this area. This underpinning knowledge is, however, challenging. For example, patients with multiple conditions or on multiple medications are often excluded from clinical trials [106]. However, EHRs afford the possibility of observational studies important for understanding multimorbid disease risks, care processes, and care outcomes. Such observational data have established value in decreasing the prescribing cascade and other iatrogenesis [107]. Automation of care pathways/processes that are poorly understood, such as merging guidelines [30], may lead to unhelpful or harmful clinical actions. The informatics challenge herein is to build the evidence base about multimorbid care while engineering more supportive/directive clinical information systems incrementally. The clinical epidemiology and health services research must be interwoven with the systems development. Gathering more clinical evidence and getting more involvement from patients and health professionals is central to finding a technological approach to managing multimorbidity and enhancing patient safety. At the same time, rigorous evaluation of all sociotechnical and human-computer interaction aspects of produced CDS interventions is certainly a priority for the future.

Patients with multiple conditions are one of the most important groups for health care systems to understand and evolve to serve [33]. There are multiple dynamics in which CDS and health informatics can contribute in meeting this challenge: (1) using EHR data to understand multimorbidity and plug a relatively sparse evidence base, (2) coproducing care decisions between patients and practitioners in the face of complexity and uncertainty, and (3) blending n-of-1 patient experiments/experience with evidence about the “average patient like Mrs X...” It is hard to conceive of such complexity being tamed by today’s EHR interfaces, punctuated by blizzards of alerts and dashboards. Future CDS may be part of an integrated health avatar [108]: “the electronic representation of an individual’s health as directly measured or inferred by statistical models or clinicians.” To achieve such integration, however, there is a pressing need for more realistically complex CDS research, particularly in multimorbidity.

## Abbreviations

EHR: electronic health record
CDS: clinical decision support
NICE: National Institute for Health and Care Excellence
